# Thrombus aspiration during primary percutaneous coronary intervention leads to reduced myocardial edema and microvascular obstruction in infarct segment post acute myocardial infarction

**DOI:** 10.1186/1532-429X-13-S1-P133

**Published:** 2011-02-02

**Authors:** Mohammad I Zia, Nilesh R Ghugre, Kim A Connelly, Graham A Wright, Alexander J Dick

**Affiliations:** 1 Sunnybrook Health Sciences Centre, Toronto, ON, Canada

## Introduction

Thrombus aspiration has been previously shown to improve myocardial blush grade during primary percutaneous coronary intervention (PCI) for acute myocardial infarction (AMI), with no impact on mortality or reinfarction. The impact of thrombus aspiration on myocardial edema and hemorrhage is unknown.

## Purpose

Our goal was to determine the impact of thrombus aspiration during primary PCI on myocardial edema and hemorrhage post AMI.

## Methods

Thirty-seven patients were enrolled post AMI and underwent CMR on a GE Signa Excite, 1.5T scanner with a 8-channel receive coil at 48 hours post MI. T2 maps were computed from a previously validated cardiac-gated spiral imaging sequence with T2 preparations yielding TEs=2.9,24.3,88.2,184.2 ms to assess myocardial edema. The T2* sequence was a multiecho acquisition with 8 echoes (between 1.4 and 12.7ms) acquired at TR=14.6ms. T2-weighted imaging using a breath-hold triple IR fast spin echo sequence and delayed hyperenhancement (DHE) were also performed. We retrospectively stratified patients into those that had received thrombus aspiration versus not, based on the operator’s discretion.

## Results

We compared 26 patients (mean age:59.4 years; 5 anterior infarcts; mean CK:2022 IU/L) that did receive thrombus aspiration versus eleven patients (mean age:58.5 years; 4 anterior infarcts; mean CK:2070 IU/L) that did not during primary PCI. The mean T2 was higher in the infarct segment (IS) compared to remote segment (RS) in both groups of patients, i.e. those that did receive thrombus aspiration (51.5 ms vs 40.9 ms; p<0.0001) versus those that did not (63.6 ms vs 41.2 ms; p<0.0001). The mean T2* was significantly lower in the IS compared to RS in both patients that did receive thrombus aspiration (25.6 ms vs 36.5 ms; p<0.0001) versus those that did not (26.3 ms vs 39.2 ms; p<0.004). The mean T2 was significantly lower in the IS of patients that did receive thrombus aspiration versus those that did not (51.5 ms vs 63.6 ms; p<0.0001). The mean T2* was equivalent in the IS of both patient groups (25.6 ms vs 26.3 ms; p=0.77). There was a lower incidence of microvascular obstruction (MVO) in the thrombus aspiration group (58% vs 91%; p=0.046).

## Conclusions

Our retrospective study demonstrates that thrombus aspiration does lead to reduced myocardial edema and MVO in the IS, compared to patients that did not receive this therapy during AMI. Thrombus aspiration seems to reduce the area at risk during myocardial infarction which may prevent deleterious left ventricular remodeling.

